# Index of consciousness monitoring during general anesthesia may effectively enhance rehabilitation in elderly patients undergoing laparoscopic urological surgery: a randomized controlled clinical trial

**DOI:** 10.1186/s12871-023-02300-z

**Published:** 2023-10-04

**Authors:** Fengling Qi, Long Fan, Chunxiu Wang, Yang Liu, Shuyi Yang, Zhen Fan, Fangfang Miao, Minhui Kan, Kunpeng Feng, Tianlong Wang

**Affiliations:** 1 Department of Anesthesiology and Operating Theatre, Xuanwu Hospital, National Clinical Research Center for Geriatric Diseases, Capital Medical University, Beijing, China; 2 Department of Anesthesiology, The First Affiliated Hospital of Baotou Medical College, Inner Mongolia University of Science and Technology, Baotou, China; 3 Department of Evidence-based Medicine, Xuanwu Hospital, National Clinical Research Center of Geriatric Diseases, Capital Medical University, Beijing, China

**Keywords:** Elderly patients, Index of consciousness monitoring, Postoperative outcome, Laparoscopic urological surgery, General anesthesia

## Abstract

**Background:**

Based on electroencephalogram (EEG) analysis, index of consciousness (IoC) monitoring is a new technique for monitoring anesthesia depth. IoC is divided into IoC_1_ (depth of sedation) and IoC_2_ (depth of analgesia). The potential for concurrent monitoring of IoC_1_ and IoC_2_ to expedite postoperative convalescence remains to be elucidated. We investigated whether combined monitoring of IoC_1_ and IoC_2_ can effectively enhances postoperative recovery compared with bispectral index (BIS) in elderly patients undergoing laparoscopic urological surgery under general anesthesia.

**Methods:**

In this prospective, controlled, double-blinded trail, 120 patients aged 65 years or older were arbitrarily assigned to either the IoC group or the control group (BIS monitoring). All patients underwent blood gas analysis at T_1_ (before anesthesia induction) and T_2_ (the end of operation). The Mini-Mental State Examination (MMSE) and Montreal Cognitive Assessment (MoCA) were administered to all patients at T_0_ (1 day before surgery) and T_4_ (7 days after surgery). Serum concentrations of C-reactive protein (CRP) and glial fibrillary acid protein (GFAP) were assessed at T_1_, T_2_, and T_3_ (24 h after surgery). Postoperative complications and the duration of hospitalization were subjected to comparative evaluation.

**Results:**

The incidence of postoperative cognitive dysfunction (POCD) was notably lower in the IoC group (10%) than in the control group (31.7%) (*P* = 0.003). Postoperative serum CRP and GFAP concentrations exhibited significant differences at time points T_2_ (CRP: *P* = 0.000; GFAP: *P* = 0.000) and T_3_ (CRP: *P* = 0.003; GFAP: *P* = 0.008). Postoperative blood glucose levels (*P* = 0.000) and the overall rate of complications (*P* = 0.037) were significantly lower in Group IoC than in Group control.

**Conclusion:**

The employment of IoC monitoring for the management of elderly surgical patients can accelerate postoperative convalescence by mitigating intraopera**t**ive stress and reducing peripheral and central inflammatory injury.

**Trial registration:**

Chinese Clinical Trial Registry Identifier: ChiCTR1900025241 (17/08/2019).

## Background

Laparoscopic surgery has developed into a mainstream surgical approach in the field of urology in recent years [1, 2]. However, the special pathophysiological conditions of elderly patients and the effect of the pneumoperitoneum on respiratory circulation during surgery may trigger a strong stress response in the body [3], which are prone to adverse prognostic outcomes such as neurocognitive disorders and damages to vital organs after surgery [4]. Therefore, precise anesthesia management is essential for enhanced recovery after surgery (ERAS). For example, without optimal anesthesia monitoring, either an inadequate or excessive dosage of anesthetics can be detrimental to vital organs. This could potentially trigger episodes of hypotension, which may lead to organ ischemic damage and dysfunction [5–7], result in delayed postoperative recovery, and increase the risk of postoperative nausea, vomiting, and neurological complications such as vertigo [8], delirium, postoperative POCD, and more [9, 10]. Furthermore, determining the intraoperative depth of anesthesia in patients who have received preoperative sedation and analgesics can pose a challenging problem. Proper monitoring in such cases can be highly beneficial in guiding the precise administration of drugs [10, 11].

Perioperative stress may induce the release of stress hormones and inflammatory factors in large amounts and consequently activate the systemic inflammatory cascade in surgical patients, thereby affecting the postoperative recovery process [12]. In the past, the determination of the patient’s anesthetic drug dosage relied to some extent on conjecture. This was rooted in the examination of the patient’s blood pressure, heart rate, the nature of the surgical procedure, and the pharmacokinetic attributes of analgesic medications, all in conjunction with individual expertise [13, 14]. Anesthesia depth monitoring based on electroencephalogram (EEG) analysis, wherein the intraoperative cerebral function is monitored, can help reduce surgical stress response, enhance patient recovery, and has been widely used in clinical anesthesia [15]. Currently, bispectral index (BIS) is the most commonly used and the only EEG approved by the Food and Drug Administration for monitoring the depth of general anesthesia [16]. However, the use of BIS in general anesthesia only reflects the depth of sedation at the cerebral cortex level. It does not account for surgical stimuli, endotracheal intubation, laryngeal mask placement, and other potentially harmful stimuli [17]. Therefore, to ensure perioperative and long-term brain health in elderly patients, there is an urgent need to establish a stable and effective evaluation system to optimize the current anesthesia management, the accuracy of anesthetic dosage, and attenuate or block various injurious stimuli to the brain [18].

Index of consciousness (IoC) monitoring (Angel-6000 A Multi-parameter Anesthesia Monitor, Shenzhen Weihao Kang Medical Technology Co., Ltd, Guangdong, China) is a novel technique based on EEG analysis for monitoring anesthesia depth. It is divided into IoC_1_ and IoC_2_, both of which range from 0 to 99. IoC_1_ is used to estimate the patient’s sedation status, which has a strong association with BIS [19]. IoC_2_ is a component separation of the acquired EEG based on IoC_1_, and the brain wave energy is calculated based on special frequency bands to accurately predict the index of pain injury [20]. The prevailing consensus suggests that an IoC_1_ within the 40–60 range signifies a state of suitable sedation, IoC_1_>60 indicates insufficient sedation, and IoC_1_ falling below 40 represents excessive sedation. As for IoC_2_, values within the 30–50 range denote an appropriate level of analgesia, IoC_2_>50 signifies inadequate analgesia, and IoC_2_<30 represents excessive analgesia. Several studies have focused on the effect of IoC_1_ on sedation depth [21, 22], while some have examined the influence of IoC_2_ on analgesia [23, 24]. However, only a few studies have assessed the simultaneous feedback modulation of IoC_1_ and IoC_2_. In the present study, we investigated whether combined monitoring of IoC_1_ and IoC_2_ can effectively enhances postoperative recovery compared with BIS in elderly patients undergoing general anesthesia in laparoscopic urological surgery.

## Methods

### Study design and settings

This prospective, randomized, controlled, double-blinded trial was approved by the institutional review board of Xuanwu Hospital, Capital Medical University (LYS [2019]051) and registered with the Chinese Clinical Trial Registry (number: ChiCTR1900025241; date of registration: 17/08/2019). The surgeries were carried out at Xuanwu Hospital in Beijing, China. All participants granted both verbal and written informed consent. This study followed the Consolidated Standards of Reporting Trials (CONSORT) reporting guideline for randomized clinical trials.

### Participants

The investigators assessed the study participants the day before surgery (or on Friday for participants scheduled for surgery the following Monday). The same surgical and anesthesia teams performed all of the procedures. The inclusion criteria for elderly patients were as follows: (1) patients undergoing elective laparoscopic urological surgery, (2) age 65–85 years old, (3) American Society of Anesthesiologists (ASA) grade I-III, (4) at least 6 years of education **(for better understanding and making judgements to scale assessment)**. The exclusion criteria were as follows: (1) severe impairment of vision, hearing, and language communication; (2) Mini-Mental State Examination (MMSE) ≤ 23; (3) change in surgical procedure following anesthesia; (4) return to the intensive care unit following surgery; (5) conditions causing severe hemodynamic fluctuations (such as severe allergic reactions or major bleeding); (6) refusal or unexpected discharge.

### Randomization and blinding

Prior to the preoperative visit, patients were assigned randomly to either the control or IoC group. A researcher, maintaining the study’s confidentiality, generated a random number table through an online software program (https://tools.medsci.cn/rand). The random allocation employed a thorough concealment technique: the grouping information within the random number table was enclosed within consecutively numbered, sealed, opaque envelopes. Upon enrolling eligible patients, the anesthetist opened the relevant envelope, and the patient received the designated treatment according to the assigned group. Patients and assessors of outcomes (comprising Neuropsychiatric Scale testers and blood sample testers) remained unaware of the group assignments.

### Study procedures

None of the patients received sedatives or anticholinergics before anesthesia induction. The following parameters were continuously monitored: blood pressure, heart rate, finger pulse oxygen saturation, partial pressure of end-tidal carbon dioxide (P_ET_CO_2_), and body temperature. For monitoring blood gas and hemodynamics, an arterial catheter was placed into the radial artery. For anesthesia induction, sufentanil 0.3–0.5 µg.kg^− 1^ and etomidate 0.2–0.3 mg.kg^− 1^ were injected intravenously until the patient lost consciousness, following which rocuronium 0.6 mg.kg^− 1^ was injected intravenously. Tracheal intubation was performed, and the patient was ventilated with a 50% O_2_-air combination at a P_ET_CO_2_ of 30–35 mmHg. The initial pumping dose for anesthesia maintenance was set as 4 mg.kg^− 1^.h^− 1^ for propofol and 0.2–0.4 µg.kg^− 1^.min^− 1^ for remifentanil. The dosage was adjusted according to the IoC range in the IoC group (IoC_1_: 40–60, IoC_2_: 30–50) and the BIS range in the control group (BIS: 40–60). For both groups, goal-directed fluid therapy was performed intraoperatively to maintain pulse pressure variation (PPV) < 13%. To maintain patients’ intraoperative blood pressure above 20% from baseline, norepinephrine was preventatively infused and regulated at a dosage of 0.03 µg.kg^− 1^.min^− 1^ to 0.10 µg.kg^− 1^.min^− 1^. The nasopharyngeal temperature was monitored and maintained between 36 and 37 °C using an intraoperative warming device. After surgery, all patients received patient-controlled intravenous analgesia (oxycodone 0.5 mg.kg^− 1^ and ondansetron 8 mg diluted to 100 mL with normal saline). A bolus dose of oxycodone 1 mg and a locking time of 5 min were set as PCIA parameters.

**Fig. 1:**
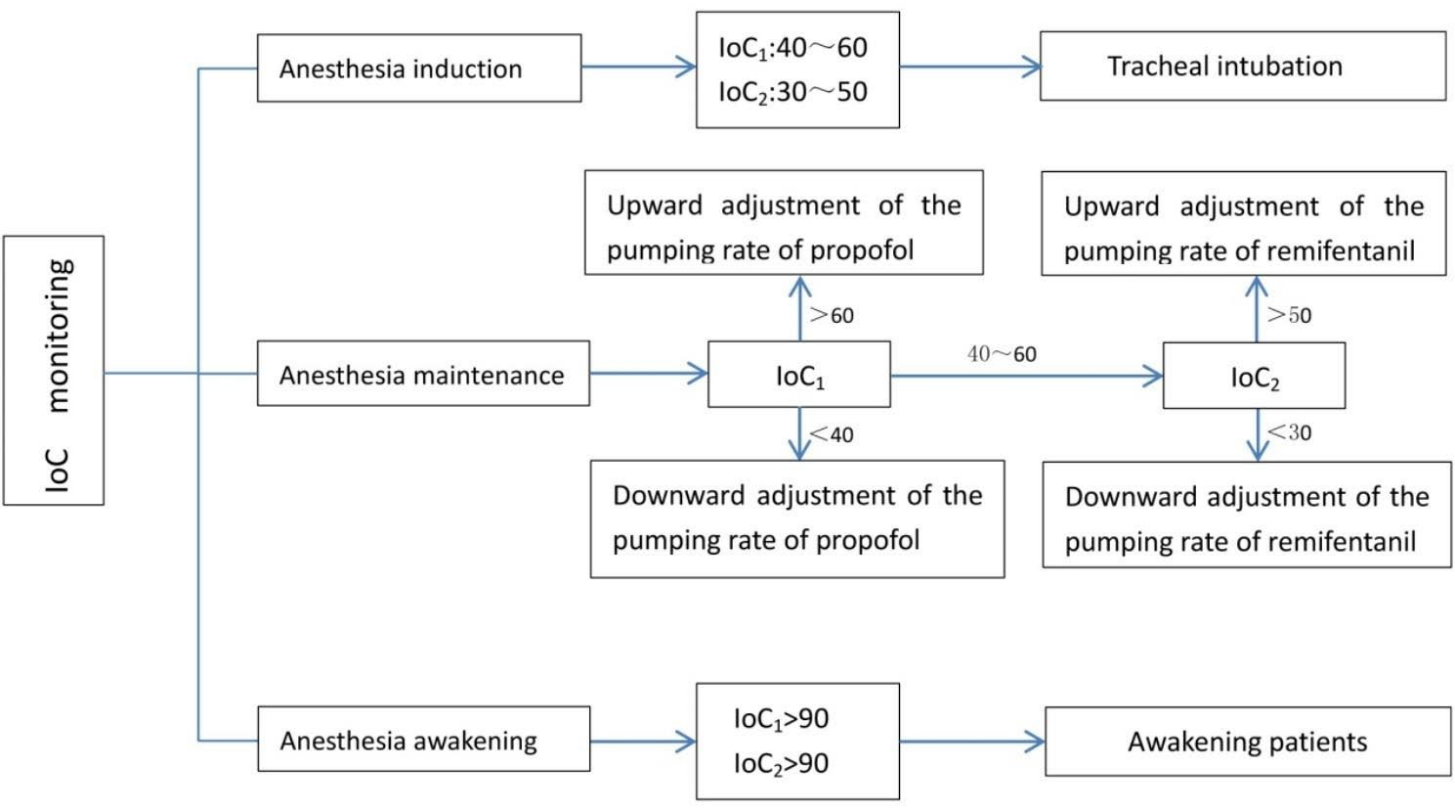
Anesthesia management of IoC group

IoC_1_ (sedation depth) and IoC_2_ (analgesia depth) monitoring was performed in the IoC group. IoC_1_ and IoC_2_ indices were set in the range of 40–60 and 30–50, respectively, during anesthesia maintenance. When the IoC_1_ index was more than 60 beyond 1 min, the propofol infusion rate was regulated by 0.4 mg.kg^− 1^.h^− 1^ increment to less than 60. When the IoC_1_ index was less than 40 for 1 min, the propofol infusion rate decreased by 0.4 mg.kg^− 1^.h^− 1^ dosage to more than 40. When the IoC_2_ index range could not be easily modulated, the IoC_1_ index was first regulated between 40 and 60 to ensure the appropriate depth of sedation. When the IoC_2_ index was more than 50 beyond 1 min, the dosage of remifentanil infusion was increased by 0.04 µg.kg^− 1^.min^− 1^ increment or bolus injection of 1 µg.kg^− 1^ was administered to reach an IoC_2_ index of less than 50 intervals. When the IoC_2_ index was less than 30 beyond 1 min, the infusion rate of remifentanil was down regulated by 0.04 µg.kg^− 1^.min^− 1^ to reach an IoC_2_ index of less than 30. Propofol and remifentanil infusion was stopped at the end of the surgery. The patient reached the awakened state when the IoC index was increased (IoC_1_ index of more than 70 and IoC_2_ index of more than 90) (Fig. 1).

Patients in the control group received the general anesthesia procedure. When the BIS index was more than 60 for 1 min, the infusion rate of propofol was increased by 0.4 mg.kg^− 1^.h^− 1^ to a BIS index of less than 60. When the BIS index was less than 40 for 1 min, the infusion rate was down regulated by 0.4 mg.kg^− 1^.h^− 1^ to a BIS index of more than 40. The infusion rate of remifentanil during anesthesia maintenance was determined by surgical stress, hemodynamic parameters, and the anesthesiologist’s experience. Propofol and remifentanil infusion was stopped at the end of the surgery. The patient reached the awakened state when the BIS index was more than 90.

Data source and collection.

The same neuropsychologist performed both MMSE and Montreal Cognitive Assessment (MoCA) assessments for patients 1 day before the surgery (T_0_) and 7 days after the surgery (T_4_). Data from the practical effect collected by the research group [25] in the past were used to calculate incidence of POCD: the practical effect was 1.92 ± 1.19, and the Z-score was calculated using the following formula: [MoCA score (T_4_) − MoCA score (T_0_) − practice effect (mean)]/practice effect (standard deviation). When the Z-score was ≥ 1.96, a diagnosis of POCD was determined.

Blood samples were collected from patients’ radial artery at T_1_ (before anesthesia induction) and T_2_ (end of the operation) for blood gas analysis. Blood gases were analyzed and measured using the standard ABL800 FLEX (Radiometer Medical, Denmark) in a standard blood gas syringe. The analysis was performed immediately after the blood samples were collected from the patient (approximately 1–3 min).

Venous blood samples (4 mL) were obtained thrice: before anesthesia induction (T_1_), end of surgery (T_2_), and 24 h after surgery (T_3_). The blood samples were centrifuged at 1,000 rpm for 15 min, and the supernatant was stored at − 80 °C. Serum levels of C-reactive protein (CRP) and glial fibrillary acidic protein (GFAP) were measured using enzyme-linked immunosorbent assay kits (CUSABIO, Wuhan, Hubei, China) according to the manufacturer’s instruction.

Demographic and clinical information, namely; age; gender; body mass index (BMI); ASA grade; education level; history of hypertension, diabetes, and coronary artery disease; and preoperative diagnosis was collected from the patients 1 day before the surgery. Additionally, the intraoperative dosage of propofol, remifentanil, and norepinephrine; anesthesia duration; pneumoperitoneum duration; Infusion volume; bleeding volume; and urine output were recorded. Postoperative complications (stroke, acute myocardial injury, acute myocardial infarction, arrhythmia, heart failure, pulmonary infection, pulmonary atelectasis, respiratory failure, pulmonary embolism, acute kidney injury, and urinary tract infection), and length of stay were also recorded.

### Primary and secondary endpoints

The primary endpoint was the incidence of POCD, which was evaluated using MoCA. The secondary endpoints included inflammatory markers (CRP and GFAP), arterial blood gas analysis (pH, PaCO_2_, blood lactic acid levels, blood glucose concentration), new postoperative complications of brain, heart, lung, kidney, and urinary tract infections, and length of hospital stay.

### Statistical analysis

The sample size was calculated to determine the estimated incidence of POCD, which is the primary outcome. In a pilot study, 30 patients who met the eligibility requirements were randomly allocated to two groups, with 15 patients in each group. In the IoC and control groups, POCD incidence was 1/15 and 4/15, respectively. The sample size was estimated using PASS15 software, and based on α = 0.05 and 1-β = 0.80, 51 patients were needed in each group. After accounting for 10% of missed visits, the final sample size for each group was estimated to be 56 cases.

SPSS Statistics 25 software was used for statistical analyses. Distributions of quantitative variables were normalized using boxplots, histograms, and Kolmogorov-Smirnov tests. Variables with normal distribution were denoted as, non-normally distributed variables were denoted as median (interquartile range), and qualitative variables were denoted as frequencies. A completely randomized design with an independent samples t-test was used to analyze parameters with a normal distribution (age; BMI; education level; dosage of sufentanil, propofol, remifentanil, and norepinephrine; pneumoperitoneum duration, and anesthesia duration; volume of infusion, bleeding, and urine; first time for post-operative exhaust; MMSE, MoCA, GFAP, CRP, serum pH, PaCO_2_, blood glucose, blood lactic acid, and length of hospital stay). Mann-Whitney U test was used to analyze the indicators of non-normal distribution (ASA grade). Qualitative indicators (gender; hypertension; diabetes; coronary artery disease; POCD; cerebral, cardiac, pulmonary, and renal complications; urinary tract infections; and total complications) were analyzed using the chi-square test. Differences were considered statistically significant at *P* ≤ 0.05.

## Results

### General characteristics of the study population

**Fig. 2:**
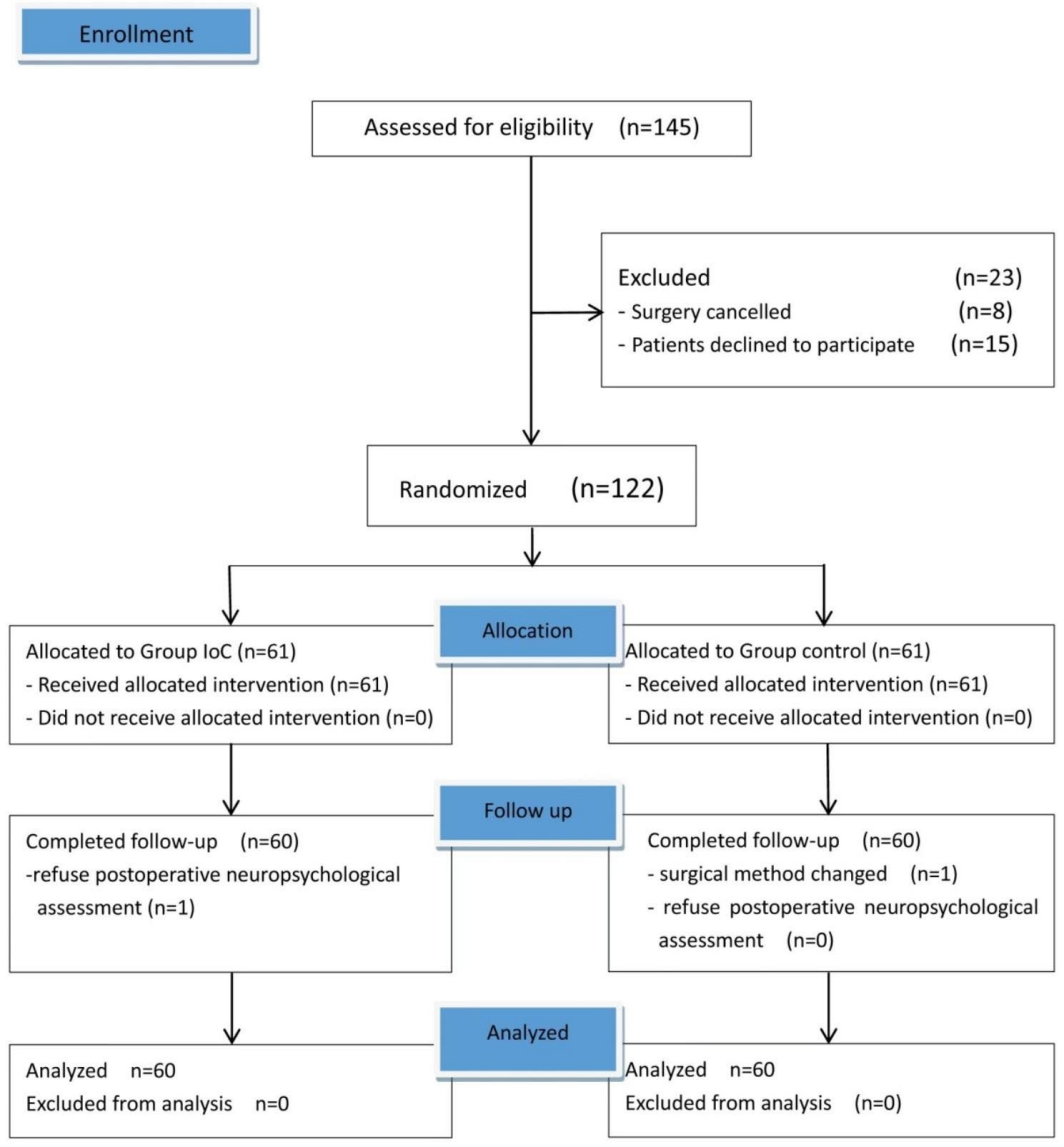
Flowchart of the study

In total, 145 elderly patients were assessed for eligibility, and 23 patients were excluded based on exclusion criteria (Fig. 2). 122 patients were randomly assigned to one of the two groups, and two elderly patients were excluded from the study (one due to refusal to undergo postoperative neuropsychological assessment in the IoC group and the other due to a change in the surgical technique in the control group). Therefore, 120 patients with a mean age of 71.2 ± 5.1 years were randomly assigned to the control and IoC groups (Table 1). Data of preoperative demographic characteristics and intraoperative variables in each group are summarized in Tables 1 and 2. No significant differences were observed in the demographic characteristics and intraoperative variables between the two groups.

**Table 1:** Demographic and clinical characteristics

Variable	Group control (n = 60)	Group IoC (n = 60)	t/Z/χ^2^-value	*P*-value	*SE* difference (95%Cl)
Age(year)	71.83 ± 4.90	70.57 ± 5.18	1.377	0.171^a^	0.92(-0.56, 3.09)
Gender (Female/Male)(n)	20/40	19/41	0.038	0.845^b^	——
BMI (kg/m^2^)	25.38 ± 2.84	24.92 ± 3.05	0.849	0.398^a^	0.54(-0.61, 1.52)
Hypertension [n (%)]	33(55.0)	34(56.7)	0.034	0.854^b^	——
Diabetes [n (%)]	20(33.3)	17(28.3)	0.352	0.553^b^	——
Coronary artery disease [n (%)]	11(18.3)	14(23.3)	0.455	0.500^b^	——
ASA classification (I/II/III)(n)	21/32/07	26/23/11	0.312	0.755^c^	——
Higher education(year)	9.88 ± 2.92	9.41 ± 2.77	0.913	0.363^a^	0.53(-0.66, 1.43)

Data are shown as mean ± SD, median [interquartile range], or number (%). BMI: Body mass index;

ASA: American Society of Anesthesiology

^a^ The *P*-value was obtained by two-sample t-test

^b^ The *P*-value was obtained by Pearson’s chi-square test

^c^ The *P*-value was obtained by Mann-Whitney U test

*P* < 0.05 was considered as statistically significant

**Table 2:** Data of perioperative variables

Variable	Group control (n = 60)	Group IoC (n = 60)	*t/*χ^2^-value	*P*-value	*SE* difference (95%Cl)
Sufentanil (µg)	33.13 ± 9.65	31.18 ± 6.60	1.292	0.199^a^	1.54(-1.09, 4.99)
Propofol (mg)	802.35 ± 418.81	673.27 ± 387.54	1.752	0.082^a^	74.29(-18.05, 276.21)
Remifentanil (mg)	4.05 ± 2.14	3.35 ± 2.12	1.800	0.074^a^	0.40(-0.11, 1.48)
Norepinephrine (mg)	6.68 ± 3.40	5.33 ± 4.36	1.891	0.060^a^	0.73(-0.08, 2.79)
Pneumoperitoneum duration (min)	150.67 ± 71.44	153.17 ± 62.89	0.203	0.839^a^	12.39(-27.03, 22.05)
Anesthesia duration (min)	220.78 ± 80.15	216.52 ± 67.51	0.315	0.753^a^	13.64(-22.74, 31.3)
Infusion volume (ml)	1423.72 ± 532.77	1377.18 ± 524.01	0.482	0.630^a^	99.84(-151.25, 244.33)
Bleeding volume (ml)	62.38 ± 64.64	64.15 ± 115.07	0.104	0.918^a^	17.18(-35.8, 32.26)
Urine volume (ml)	545.58 ± 307.57	481.43 ± 273.75	1.207	0.230^a^	56.36(-28.11, 195.2)
First time for post-operative exhaust (h)	25.73 ± 13.55	27.45 ± 13.74	0.689	0.492^a^	2.57(-7.4, 2.79)
Nausea and vomiting [n (%)]	8(13.3)	7(11.7)	0.076	0.783^b^	——

Data are shown as mean ± SD or number (%)

^a^ The *P*-value was obtained by two-sample t-test

^b^ The *P*-value was obtained by Pearson’s chi-square test

*P* < 0.05 was considered as statistically significant

### Primary endpoint

The MMSE and MoCA scores at T_0_ (1 day before the surgery) and T_4_ (7 days after the surgery) and the incidence of POCD are listed in Table 3. There were no significant differences between the two groups for preoperative MMSE and MoCA scores. The MoCA score was significantly higher in the IoC group vs. the control group (25.43 ± 2.65 vs. 24.37 ± 1.93; *P* = 0.013) at T_4_. The incidence of POCD in the IoC group was significantly lower than that in the control group (19(31.7%) vs. 6(10%); *P* = 0.003).

**Table 3:** Neuropsychological assessment and incidence of POCD

variable	Group control (n = 60)	Group IoC (n = 60)	t-value	*P*-value	*SE* difference (95%Cl)
MMSE score(T_0_)	27.53 ± 0.75	27.57 ± 0.91	0.186	0.872^a^	0.15(-0.33, 0.27)
MMSE score (T_4_)	27.75 ± 1.20	27.83 ± 1.25	0.370	0.711^a^	0.23(-0.53, 0.36)
MoCA score(T_0_)	23.62 ± 2.78	23.73 ± 3.30	0.209	0.834^a^	0.56(-1.22, 0.99)
MoCA score(T_4_)	24.37 ± 1.93	25.43 ± 2.65	2.523	0.013^a^	0.42(-1.90, -0.23)
POCD[n (%)]	19(31.7)	6(10.0)	8.539	0.003^b^	——

Data are shown as mean ± SD or number (%).MMSE: Mini-Mental State Examination; MoCA: Montreal Cognitive Assessment; POCD: postoperative cognitive dysfunction;T_0_ : 1 day before surgery; T_4_ : 7 days after surgery

^a^ The *P*-value was obtained by two-sample t-test

^b^ The *P*-value was obtained by Pearson’s chi-square test

*P* < 0.05 was considered as statistically significant

### Secondary endpoint

**Fig. 3:**
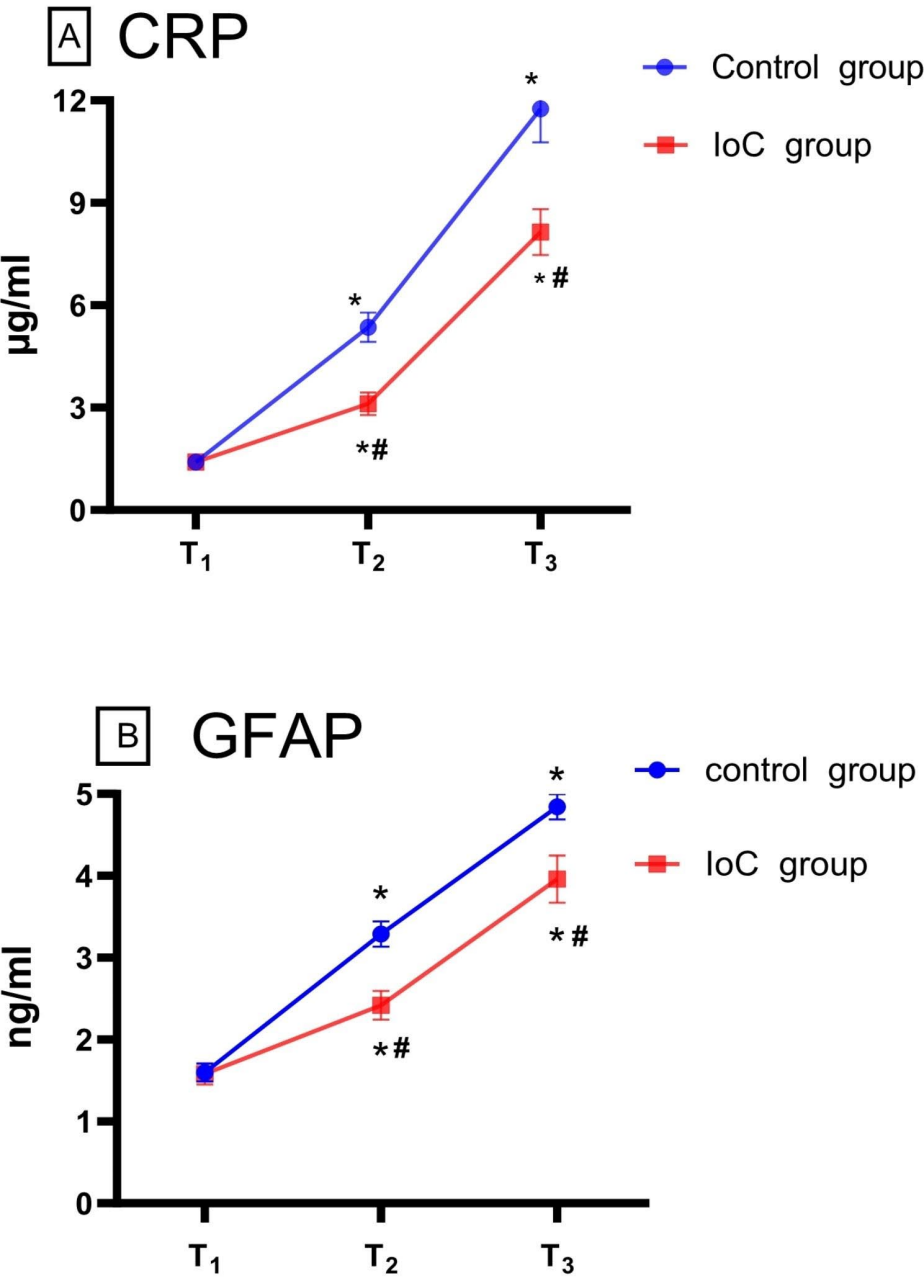
CRP and GFAP concentrations over time ∗*P* < 0.05 from T_1_ in the IoC group and the control group (statistically significant), #*P* < 0.05 from the control group (statistically significant). Abbreviation: CRP, C-reactive protein; GFAP, glial fibrillary acidic protein; T_1_: before anesthesia induction; T_2_: end of surgery; T_3_: 24 h after surgery

Serum CRP concentrations were significantly increased at the end of surgery and on postoperative day 1 in both groups. However, serum CRP levels were significantly lower in the IoC group than in the control group at the end of surgery (5.36 ± 3.36 vs. 3.11 ± 2.62; *P* = 0.000) and 24 h after the surgery (11.75 ± 7.52 vs. 8.15 ± 5.24; *P* = 0.003) (Fig. 3).

Serum GFAP levels were increased at the end of surgery and on postoperative day 1 in both groups. However, serum GFAP concentrations were significantly lower in the IoC group than in the control group at the end of surgery (3.29 ± 1.22 vs. 2.42 ± 1.38; *P* = 0.000) and 24 h after surgery (4.84 ± 1.20 vs. 3.96 ± 2.23; *P* = 0.008) (Fig. 3).

Although there was no significant difference in pH, PaCO_2_, or blood lactic acid levels between the two groups, the blood glucose concentration at the end of surgery in the IoC group was obviously lower than that in the control group (4.84 ± 1.20 vs. 3.96 ± 2.23; *P* = 0 0.000) (Table 4).

**Table 4:** perioperative arterial blood gas analysis

	Group control (n = 60)	Group IoC (n = 60)	t-value	*P*-value	*SE* difference (95%Cl)
pH					
Pre-operative	7.43 ± 0.03	7.43 ± 0.02	0.146	0.889^a^	0.01(-0.01, 0.01)
Postoperative	7.32 ± 0.05	7.31 ± 0.05	0.241	0.814^a^	0.01(-0.02, 0.02)
PaCO_2_(mmHg)					
Pre-operative	36.69 ± 3.44	37.80 ± 3.14	1.846	0.069^a^	0.6(-2.29, 0.09)
Postoperative	45.96 ± 5.78	46.45 ± 5.78	0.425	0.677^a^	1.19(-2.84, 1.85)
Blood glucose(mmol/L)					
Pre-operative	6.93 ± 1.46	6.70 ± 1.41	0.872	0.385^a^	0.26(-0.29, 0.75)
Postoperative	9.17 ± 2.04	7.11 ± 1.35	6.523	0.000^a^	0.32(1.44, 2.69)
Blood lactic acid(mmol/L)					
Pre-operative	1.52 ± 0.54	1.35 ± 0.65	1.599	0.113^a^	0.11(-0.04, 0.39)
Postoperative	1.23 ± 0.41	1.24 ± 1.24	0.071	0.945^a^	0.17(-0.35, 0.32)

Data are shown as mean ± SD.

^a^ The *P*-value was obtained by two-sample t-test

*P* < 0.05 was considered as statistically significant

No significant difference was noted in the incidence of serious postoperative complications of the brain, heart, lung, kidney, and urinary tract infections between the two groups, but the total rate of complications (observed at 7 days after the surgery) in the control group was significantly higher than that in the IoC group, with a significant difference (16(26.7%) vs. 7(11.7%), *P* = 0 0.037). The mean length of hospital stay was over 2 days shorter in the IoC group than in the control group, with an insignificant difference (Table 5).

**Table 5:** Postoperative outcomes

	Group control (n = 60)	Group IoC (n = 60)	*t/*χ^2^-value	*P*-value	*SE* difference (95%Cl)
Cerebral complications [n (%)]	0	0	——	——	——
Cardiac complications [n (%)]	2(3.3)	1(1.7)	0.000	1.000^b^	——
Pulmonary complications [n (%)]	1(1.7)	0(1.7)	——	1.000^b^	——
Renal complications [n (%)]	4(6.7)	1(1.7)	0.835	0.361^b^	——
Urinary tract infection [n (%)]	9(13.3)	5(8.3)	1.294	0.255^b^	——
Total complications [n (%)]	16(26.7)	7(11.7)	4.357	0.037^b^	——
Hospital stays(days)	15.02 ± 7.42	13.10 ± 5.13	1.649	0.102^a^	4.42(-5.42, 9.36)

Data are shown as mean ± SD or number (%)

^a^ The *P*-value was obtained by two-sample t-test

^b^ The *P*-value was obtained by Pearson’s chi-square test

*P* < 0.05 was considered as statistically significant

## Discussion

In this study, we found that anesthesia management based on IoC monitoring significantly improved post-operative neuropsychological assessment scores and had a lower total rate of complication compared to routine anesthesia management in elderly patients undergoing laparoscopic urological surgery. Although there was no significant difference in the length of hospital stay between the two groups, the number of days in the IoC group was significantly less than in the control group.

Presently, most studies pertaining to the level of analgesia predominantly focus on the exploration and advancement of monitoring techniques such as analgesia and nociception index (ANI), surgical Pleth index (SPI), skin conductance (SC), tip perfusion index (TPI) and pain-related evoked potential (PREP). Although these techniques have substantially enhanced clinical anesthesia, they do not entirely align with clinical requirements and exhibit limited applications. Studies have revealed that noxious stimuli can induce alterations in electroencephalography (EEG) [26]. In the case of children undergoing general anesthesia, the administration of noxious stimuli results in a noteworthy increase in δ-wave activity, with the response magnitude escalating in accordance with the stimulus intensity [27]. Employing EEG-based sedation monitoring for patients throughout anesthesia and surgery improves cognitive function in postoperative patients [28].

POCD is a serious neurological complication that occurs in elderly patients after surgery, which can seriously affect patients’ learning and memory abilities, survival rate 1 year after surgery, risk of dementia, length of hospital stay of patients, and socioeconomic burden [29, 30]. In our previous study, the incidence of POCD had reached 43% in elderly patients undergoing spinal surgery [31]. Inadequate sedation has become a risk factor for POCD. In Patients over 60 years old undergoing abdominal surgery, the incidence of POCD was lower when BIS value was maintained at 30–45 than that of BIS at 45–60 [32]. However, inadequate analgesia can also cause EEG changes [26]. Some studies have shown that perioperative pain can lead to POCD [33]. Therefore, we employed EEG-based IoC monitoring. The results showed that patients with anesthetic management based on IoC monitoring had significantly higher postoperative MoCA scores than the control group. MoCA has a high sensitivity and specificity for detecting mild cognitive changes and is thus used to assess cognitive function [34]. The Z score recommended by the International Study of Postoperative Cognitive Dysfunction is used to diagnose POCD [35]. Although there was no difference in total anesthetic drug dosage between the two groups, individualizing patients at different sedation and analgesia depth levels using IoC_1_ and IoC_2_ effectively reduced stress response and improved postoperative cognitive function.

A previous study also demonstrated that sustained excessive stress response might exacerbate the inflammatory response [36]. Biomarkers of inflammation and stress (e.g. CRP and cortisol) were negatively correlated with neuropsychological test assessment scores [37]. CRP is the mediator of the body’s acute phase response to tissue damage and is a sensitive indicator of the degree of surgical stress [38]. Patients with POCD have elevated CRP levels after undergoing laparoscopic cystectomy [39], and lumbar discectomy [40]. In this study, elevated levels of postoperative serum CRP indicated a systemic inflammatory response. The difference between the groups suggested that precise intraoperative anesthesia management through IoC monitoring may be effective in reducing early postoperative stress. Additionally, precise sedation and analgesia management during surgery have an immunoprotective effect on the organism [41], the present study found that IoC monitoring impairs stress-induced inflammatory response.

GFAP is a specific marker protein for astrocytes, which is the main immune effector cell of the central nervous system (CNS) and is associated with CNS neuroinflammation [42]. GFAP is rarely expressed in the brain cells under physiological conditions and shows enhanced reactive expression under pathological conditions [43]. The peripheral inflammatory response occurring after surgical anesthesia activates the CNS inflammatory response through multiple mechanisms [44], thereby stimulating the astrocytes on the blood-brain barrier to release large amounts of central inflammatory factors [45], and the resultant concentration of GFAP in the plasma significantly increases [46]. Cao et al. discovered increased GFAP expression in the hippocampus of rats with cognitive dysfunction on postoperative days 1 and 3 by testing the cognitive performance experimentally in elderly rats undergoing partial hepatectomy [47]. The present study showed that postoperative serum GFAP concentrations were lower in the IoC group than in the control group, implying that EEG-based IoC monitoring obviously reduced intraoperative brain noxiousness through individualized sedation and analgesia modulation for stress.

After a perioperative stressful event such as surgical trauma or pain, the body activates both the fast response system and slow response system (SRS). When the fast response system is not effectively controlled, the SRS is characterized by the activation of the hypothalamus-pituitary-adrenal cortex axis becomes activated. The main manifestation of SRS was insulin resistance (IR), wherein the target organs, tissues, or cells showed reduced sensitivity and responsiveness to insulin. IR is characterized by transient and reversible hyperglycemia, a condition also known as stressful hyperglycemia [48, 49]. Recent studies [50, 51] have shown that the incidence of postoperative hyperglycemia ranges from 20 to 40% in patients undergoing non-cardiac surgery. The presence of stress hyperglycemia is often accompanied by perioperative morbidity and mortality, a higher risk of postoperative wound infection, and a longer length of hospital stay. Hopkins et al. implemented standardized postoperative glycemic control management for perioperative patients and reported a 55% reduction in the incidence of postoperative wound infection [52]. Therefore, perioperative glycemic control has become an important part of anti-stress management. The present study showed that postoperative blood glucose levels and total rate of complications were significantly lower in the IoC group than in the control group. Although no significant difference was noted in the length of hospital stay between the two groups, the number of days in the hospital was significantly shorter in the IoC group than in the control group. The study findings suggest that intraoperative anesthesia management based on IoC monitoring can effectively reduce the stress response and help maintain physical homeostasis, which is conducive to rapid postoperative recovery.

Further investigation is needed to determine whether IoC_1_ and IoC_2_ monitoring can serve as indices for assessing sleep depth and the effectiveness of melatonin and other medications in treating sleep disorders [53].

## Limitations

This study has several limitations. Initially, as a single-center trial, the potential for generalizing the findings may be restricted. All the enrolled patients underwent laparoscopic urological surgery, and whether our results can be generalized to other types of surgery remains to be determined. Second, the sample size was modest, which may explain why several secondary outcome indicators showed no differences. Third, no correlation was noted between the indications of long-term recovery (particularly long-term mortality) and depth of anesthesia. Lastly, several confounding factors that could impact our results have not been accounted for, such as electrocoagulation electrosurgery induced high frequency interfering signal.

## Conclusions

In conclusion, anesthesia management based on IoC monitoring may precisely and effectively modulate intraoperative sedation and analgesia depth and significantly reduce the total rate of complications in elderly patients undergoing laparoscopic urological surgery, especially the incidence of POCD. The potential underlying mechanism may be related to the effective control of the input perception of the brain to peripheral noxious stress and inhibition of systemic inflammation and neuroinflammation. Our discoveries lay the groundwork for improved management of swift recuperation in elderly patients undergoing surgical procedures. IoC monitoring, grounded in EEG analysis, may emerge as a novel approach to monitoring and customizing sedation and analgesia. This could prove especially advantageous for elderly individuals, critically ill patients, and others necessitating personalized adjustments to anesthetic dosages.

## Data Availability

The datasets used and/or analyzed during this study are available from the corresponding author on reasonable request.

## Abbreviations

IoC: Index of consciousness
EEG: electroencephalogram
POCD: postoperative cognitive dysfunction
ERAS: enhanced recovery after surgery
BIS: bispectral index
ASA: American Society of Anesthesiologists
MMSE: Mini-Mental State Examination
MoCA: Montreal Cognitive Assessment
PETCO_2_: end-tidal carbon dioxide
PPV: pulse pressure variation
PCIA: patient-controlled intravenous analgesia
CRP: C-reactive protein
GFAP: glial fibrillary acidic protein
BMI: body mass index
CNS: central nervous system
SRS: slow response system
IR: insulin resistance
